# Nutritional Management of Patients With Enterocutaneous Fistulas: Practice and Progression

**DOI:** 10.3389/fnut.2020.564379

**Published:** 2020-10-06

**Authors:** Qin-qing Tang, Zhi-wu Hong, Hua-jian Ren, Lei Wu, Ge-fei Wang, Guo-sheng Gu, Jun Chen, Tao Zheng, Xiu-wen Wu, Jian-an Ren, Jie-shou Li

**Affiliations:** ^1^Jinling Hospital Research Institute of General Surgery, Nanjing, China; ^2^Laboratory for Trauma and Surgical Infections, Jinling Hospital, Nanjing, China; ^3^Department of General Surgery, First Affiliated Hospital of Anhui Medical University, Hefei, China

**Keywords:** enteral nutrition, enteroatmospheric fistula, enterocutaneous fistula, fistuloclysis, parenteral nutrition

## Abstract

The management of enterocutaneous fistulas (ECF) can be challenging because of massive fluid loss, which can lead to electrolyte imbalance, severe dehydration, malnutrition and sepsis. Nutritional support plays a key role in the management and successful closure of ECF. The principle of nutritional support for patients with ECF should be giving enteral nutrition (EN) priority, supplemented by parenteral nutrition if necessary. Although total parenteral nutrition (TPN) may be indicated, use of enteral feeding should be advocated as early as possible if patients are tolerant to it, which can protect gut mucosal barrier and prevent bacterial translocation. A variety of methods of enteral nutrition have been developed such as fistuloclysis and relay perfusion. ECF can also be occluded by special devices and then EN can be implemented, including fibrin glue application, Over-The-Scope Clip placement and three-dimensional (3D)-printed patient-personalized fistula stent implantation. However, those above should not be conducted in acute fistulas, because tissues are edematous and perforation could easily occur.

## Introduction

**Figure 1:**
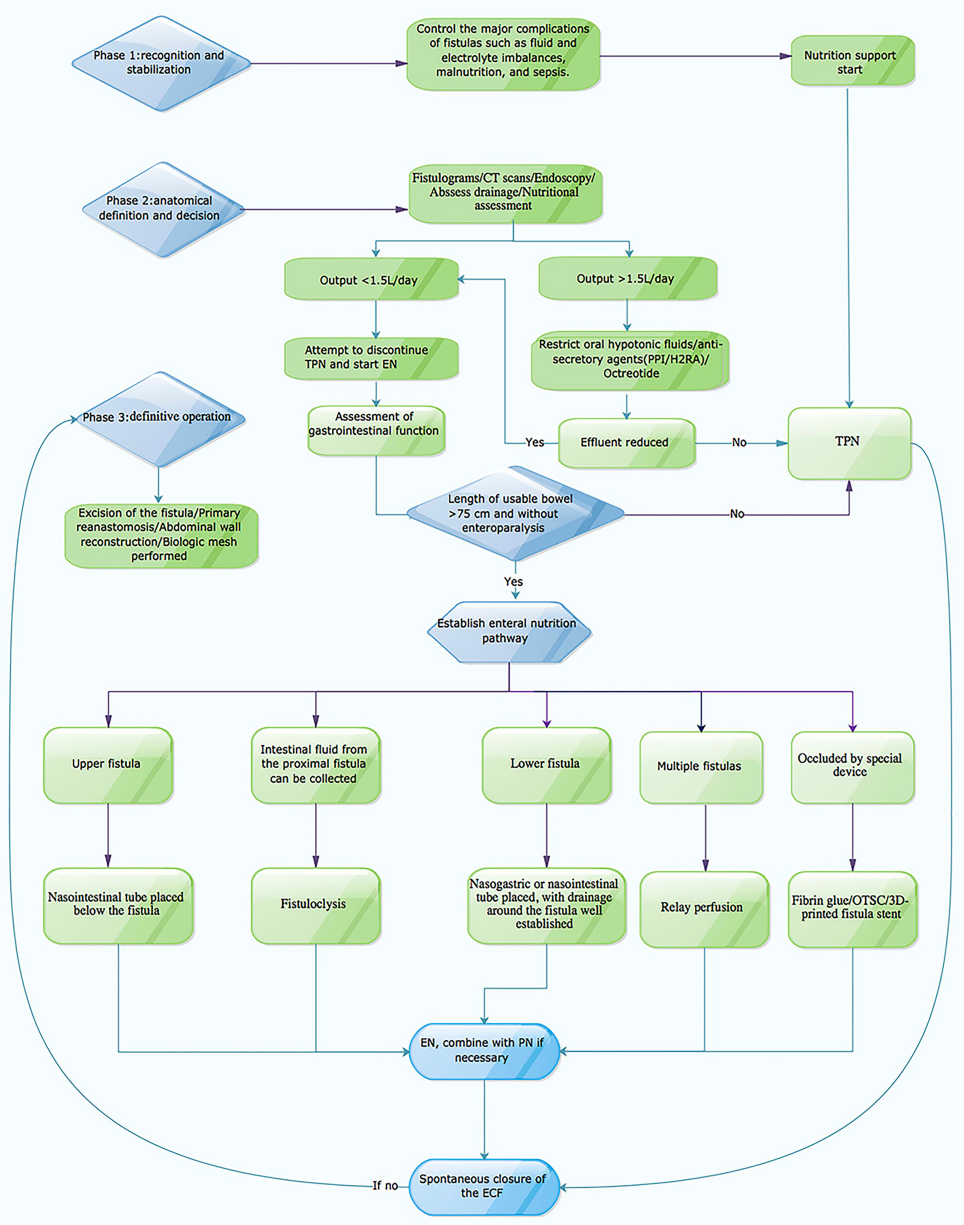
Figure showing clinical and nutritional management algorithm of ECF.

An enterocutaneous fistula (ECF) is an abnormal connection between the gastrointestinal tract and the skin or atmosphere (enteroatmospheric fistula [EAF]) (1). Sepsis, malnutrition, and electrolyte abnormalities are the classic triad of ECF complications, among which malnutrition and sepsis are the leading causes of death (2). Historically, the mortality rate for ECF patients has been as high as 40%, but has reduced significantly in the past decade to 3.5–19% (3–5). The management of ECFs is still one of the most challenging surgical problems nowadays despite great advances in surgical critical and care (Figure 1).

EAF is a subgroup of ECF and appears in open abdomen (6). EAFs are still classified as ECFs in many cases. The extensive application of damage control and abdominal opening in trauma and emergency surgery lead to EAF and challenges surgeons. EAF are difficult to manage because there is neither skin nor soft tissue surrounding or overlying the opening in the bowel.

**Figure 2:**
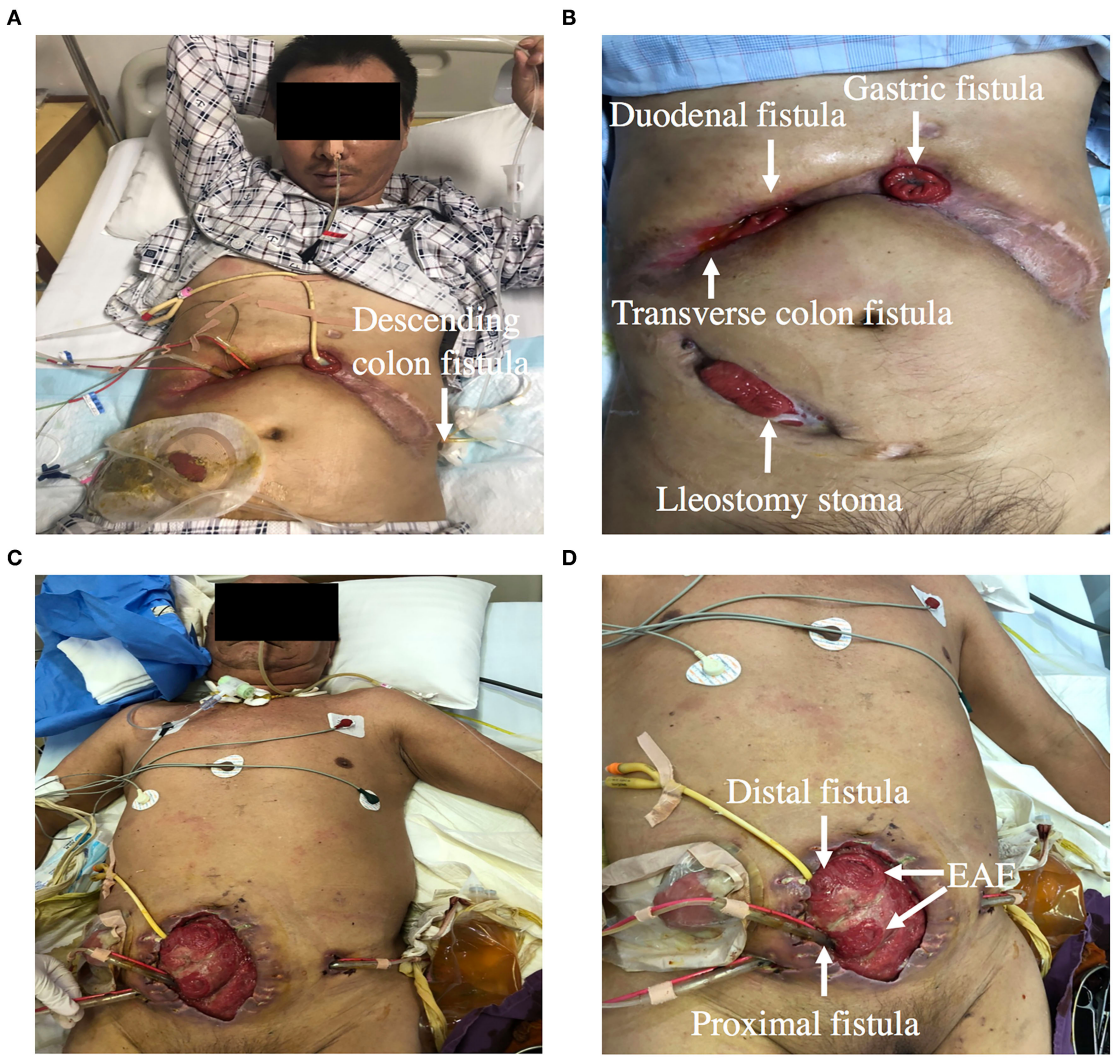
**(A,B)** Showing a patient with gastric, duodenal, transverse and descending colon fistula. **(C,D)** Showing a patient with EAF. Double-pipe was placed into proximal intestine to collect intestinal fluid, and urethral catheter was placed into distal intestine for distal feeding (fistuloclysis).

ECF and EAF are difficult complications that occur spontaneously or primarily after abdominal surgery. ECF and EAF are associated with malnutrition and sepsis (7). Due to the significant mortality and morbidity, a multi-disciplinary approach, which includes surgical, nutritional, pharmacotherapeutic interventions, as well as nurse specialists are required in the management of ECF (8) (Figure 2).

ECF/EAF patients usually have type 2 intestinal failure. Sepsis-Nutrition-Anatomy-Plan (SNAP) approach has been widely adopted. The emphasis is on the control of sepsis (S), the optimization of nutritional status (N), the understanding of the anatomy of fistula (A), and the planning of surgical treatment of fistula (P) (9, 10). ECFs are less likely to heal spontaneously in the presence of sepsis. Aggressive nutritional support cannot be successful until sepsis is treated because of impaired intestinal function in patients with sepsis. Weight and BMI provide simple and objective measurements, but caution is needed in patients with fluid imbalance and severe malnutrition. Body composition studies are particularly important for ECF patients undergoing surgical repair to ensure proper timing of surgery and to provide the best opportunity for wound healing and sepsis-free recovery. Computed tomography (CT)–measured psoas muscle density is an important predictor of poor outcomes in ECF repair. Psoas density correlates with malnutrition and frailty. This identifies ECF patients at increased risk and may benefit from additional intervention and recovery time prior to surgical repair (11).

Fragkos et al. (12) investigated the correlation between total adipose tissue area (TFA) and body mass index (BMI), various biochemical parameters, nutritional support needs, and survival in patients with ECF repair using computed tomography and magnetic resonance imaging radiology tests. The results showed that patients in the low TFA group had a higher use of parenteral nutrition. Patients receiving artificial nutrition support had a longer hospital stay. In the multivariate analysis, only age >60 years [hazard ratio (HR) 2.69, *P* < 0.02) and parenteral nutrition use (HR, 3.90, *P* < 0.02) were associated with poorer overall survival.

All ECF patients should undergo detailed anatomical assessment, including oral and enema studies, as well as fistulograms. Long-term plans for patients with intestinal failure can only be developed when progress is made in addressing infection and improving nutritional status, as the persistence of sepsis and malnutrition will prove to be the major cause of morbidity and mortality.

Malnutrition is a major determinant of negative clinical outcomes in ECF patients. Malnutrition in ECF patients increases the risk of adverse outcomes, including medical-related infections, sepsis, and intra-abdominal abscess (13). At present, providing optimal nutrition through the enteral or parenteral pathways is a mandatory component of the perioperative period for ECF patients. The use of EN and PN increases the rate of spontaneous closure and reduces mortality in ECF patients. However, the complications of TPN and EN as well as the pathways are still worthy of attention. This present review focuses on the clinical and nutritional management in ECF patients, especially nutritional management.

## Clinical Management of ECF

Our approach to ECF conforms to the three-phase approach described by Schecter et al. (1). The first phase is recognition and stabilization, the initial imperative is stabilization of the patient as soon as an ECF is recognized. The goal of stabilizing ECF fistulas is to control fluid and electrolyte imbalances, malnutrition, sepsis, abscess formation and wound infection. The stabilization phase must proceed rapidly because these problems are associated with morbidity and mortality. It is best to address these problems within 24–48 h after the fistula is recognized.

**Figure 3:**
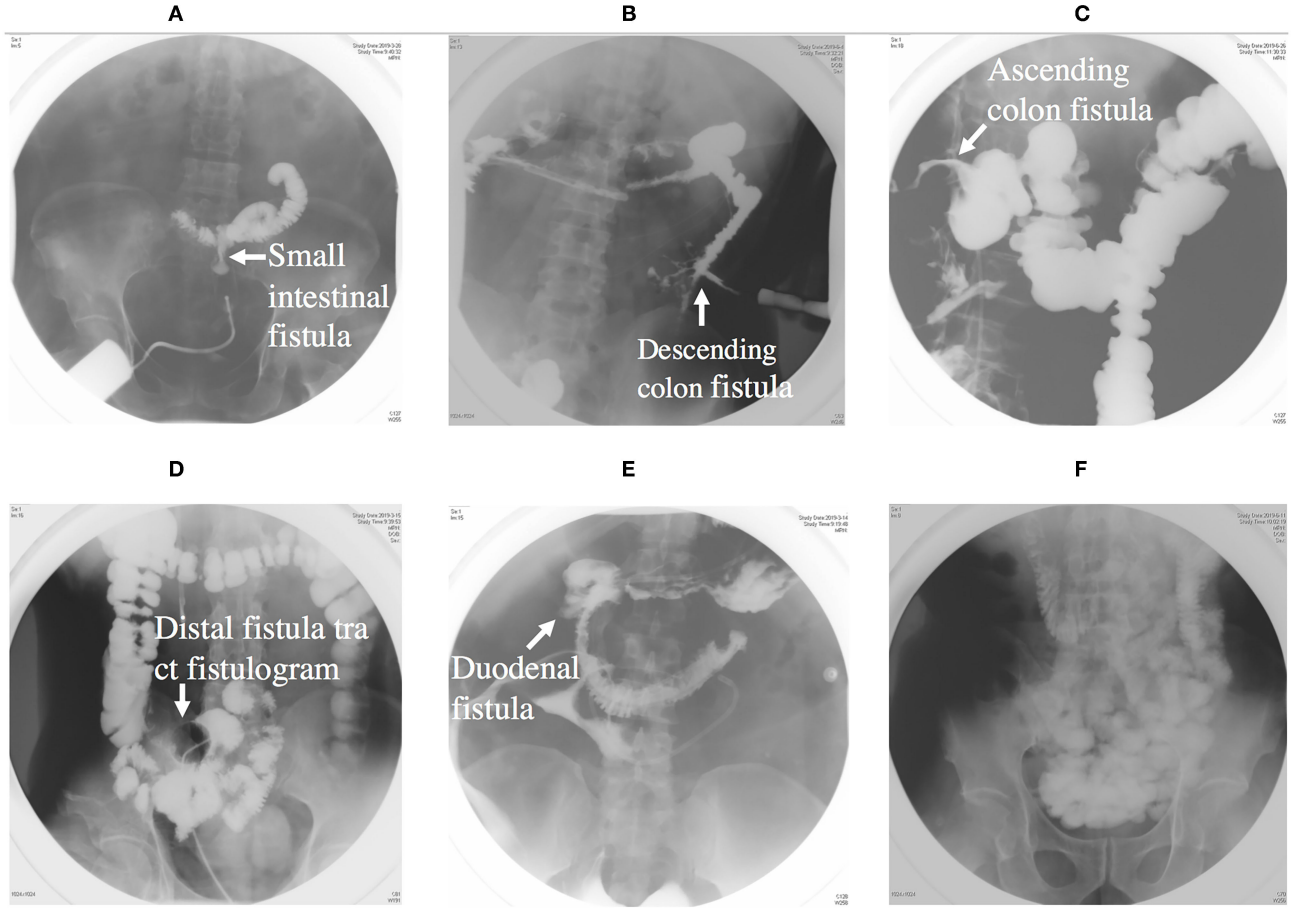
**(A)** Showing an intestinal fistula through fistula tract imaging. **(B)** Showing a descending colon fistula. **(C)** Showing an ascending colon fistula. **(D)** Showing distal intestinal tract of fistula. **(E)** Showing a duodenal fistula. **(F)** Showing proximal intestinal tract.

**Figure 4:**
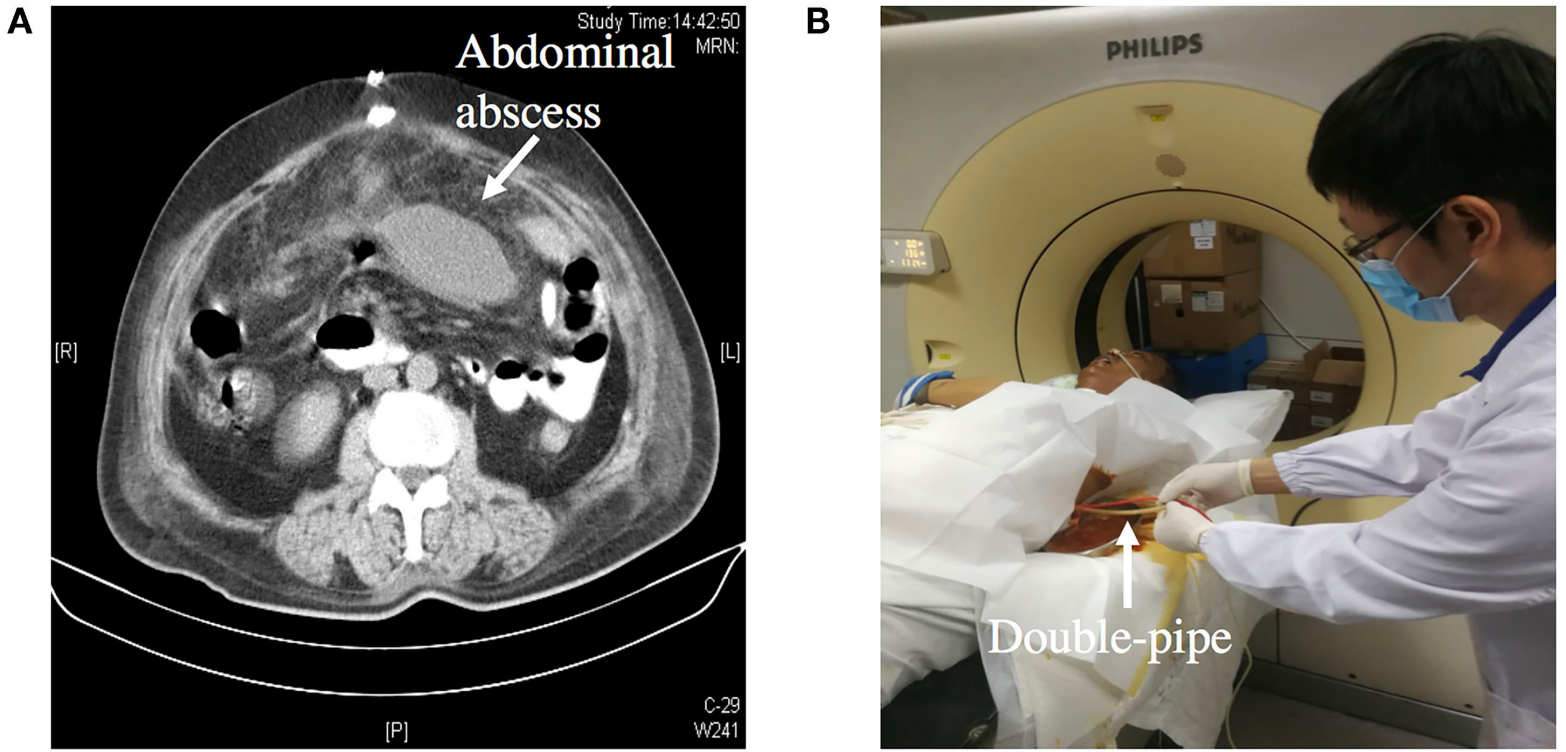
**(A)** Showing an abdominal abscess due to ECF. **(B)** Showing CT-guided double-pipe placement for continuous irrigation and drainage of the abscess.

The next phase is anatomical definition and decision, during which fistulograms (Figure 3) and CT scans are performed during this period, and endoscopy is performed if necessary. Fistulograms is very important for the diagnosis of ECF. It can determine the location and length of fistula, whether it is a single fistula or multiple fistulas, and how far it is from pylorus, ileocecum, and anus. CT scan can help to determine whether there is abscess in the abdominal cavity and intestinal obstruction. Source control may be obtained with percutaneous drainage of an abscess visualized on CT scan (Figure 4). For the majority of ECF patients, fistulograms and CT are enough for diagnosis.

The effluent nature and output probability are highly correlated to the anatomic region. Low, moderate, and high output fistula are generally defined as <200 mL/day, 200–500 mL/day, and >500 mL/ day, respectively (14). Low-output fistula has a higher likelihood of spontaneous closure, and a portion of patients with ECF will heal spontaneously with appropriate nutritional support and wound care (15). The purpose of describing fistula anatomy is to evaluate the spontaneous closure possibility. A final operation may be considered if the fistula fails to respond to medication within 4–6 weeks.

Major abdominal surgery results in dense peritoneal reactions. The reaction usually last 1–6 weeks, and begin to resolve 6 weeks later (16). Surgery performed within this window usually results in additional bowel resection, intestinal vascular disconnection, and greater damage. During this period, ECFs should be limited to control abdominal abscess, intestinal gangrene, peritonitis and sepsis.

Fortunately, up to one third of all ECFs will close spontaneously, whereas most EAFs will require surgery for definitive closure (17, 18). The surgeon must also recognize the tremendous catabolism that occurs with peritonitis and a huge open wound and treat with appropriate nutrition support. This often requires a combination of EN and PN (6).

**Figure 5:**
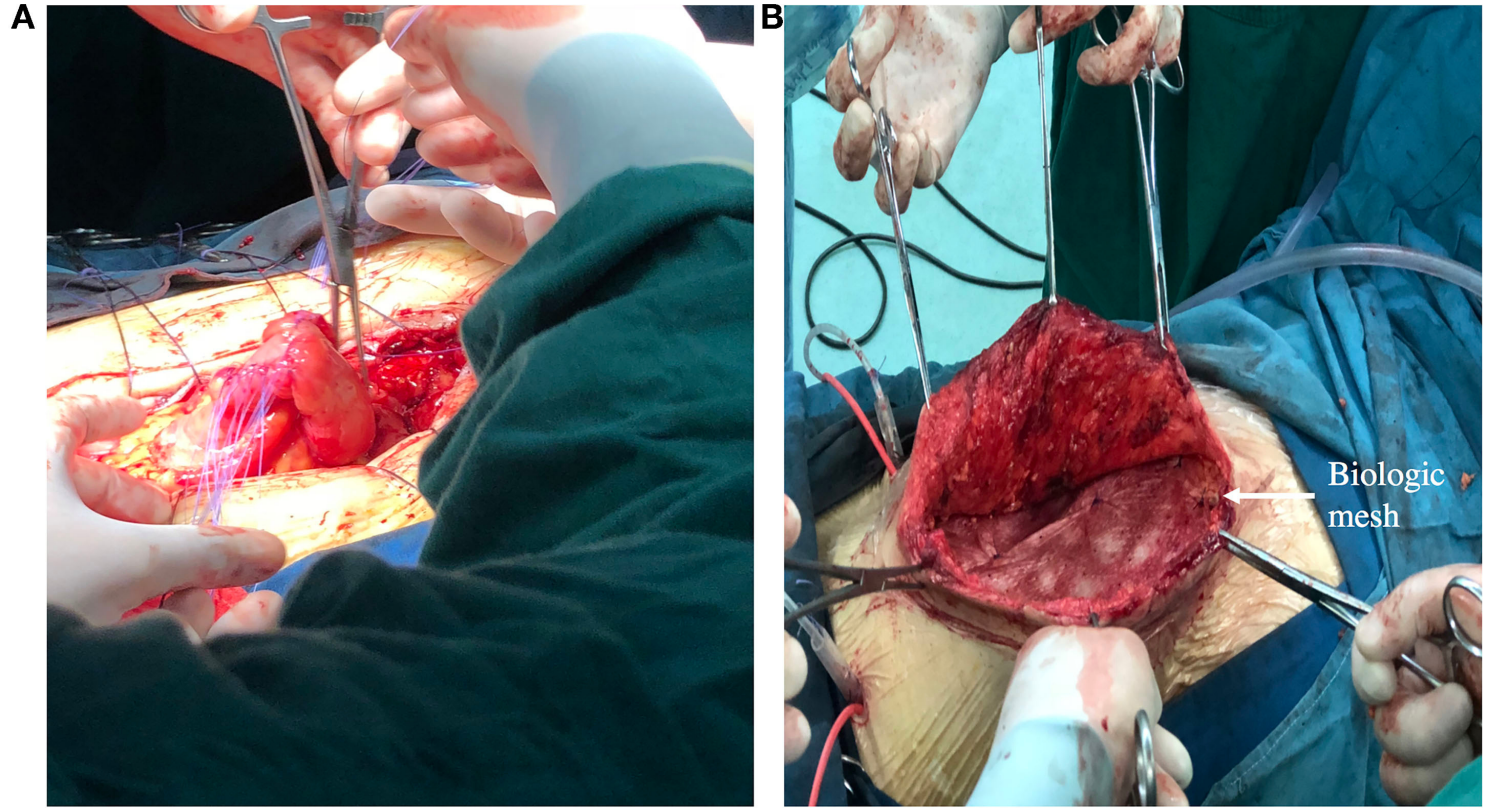
**(A)** Showing a definitive operation that the segment of bowel where fistula resided was resected and intestinal tract continuity was reestablished. **(B)** Showing definitive reconstruction of the abdominal wall defect with biologic mesh.

In the final phase, definitive operation, the patient underwent fistula resection and primary anastomosis. If necessary, abdominal wall reconstruction and biologic mesh are also performed in this phase (Figure 5). The decision of operation is based on a variety of factors, such as the patient's overall health and preference, fistula location and effluent. The following patients should be considered for definitive surgery: (1) eversion of the mucosa of the fistula; (2) the fistula has not closed spontaneous within 30 days; (3) those conditions exist such as distal obstruction, inflammatory bowel disease, neoplasm, radiation enteritis. According to our practice in the Fistula Treatment Center of Jinling Hospital of China, attempts should made to defer operation for at least 3 months. Before operation, BMI index of the patients should >18.5 and the patients need to blow at least 40 balloons diameter at 5 cm per day (at least 1–2 weeks), in order to reduce the incidence of post-operative pneumonia and atelectasis.

## Nutritional Management of ECF

ECF patients often develop malnutrition during their medical treatment and nutritional support plays a vital role. Correcting and preventing further malnutrition is a clinical challenge for multidisciplinary medical teams and patients (14). In 1978, Prof. Jie-shou Li of Jingling Hospital put forward the principle of nutritional support for patients with ECF, which is giving enteral nutrition priority, supplemented by parenteral nutrition (19).

Traditional ECF management includes TPN and avoiding EN to minimize fistula output. TPN has been shown to reduce gastrointestinal secretions, which is vital to managing high output fistula. The role of TPN support in ECF management is to prevent further deterioration of malnutrition, thereby preventing further deterioration of the ECF patients. Therefore, it is considered to have a major therapeutic effect. The nutritional status, closure rate and survival rate of fistula patients were improved with the introduction of TPN in 1970's. Over the past 40 years, TPN has provided a cornerstone of therapy for nutrition support. TPN not only reverses the catabolic state of ECF patients, but also allows ECFs time to heal spontaneously. Those who persisted can be closed by surgery with infection-free and has a good chance of success.

Nutritional status plays an important role on clinical outcome of ECF patients. Optimal nutritional support is closely related to the mortality rate and spontaneous fistula closure. Those patients who received 1,500–2,000 calories per day had a lower mortality rate and a higher fistula closure rate compared with the patients who received <1,000 calories per day (20, 21). Nitrogen balance is clinically acceptable as an indicator of anabolic status. Negative nitrogen balance indicates that the nutrition plan need to be modified. It is necessary to include nitrogen balance calculation in the nutrition management of ECF patients because of the protein loss in the fistula output (22). A positive nitrogen balance indicates that the patient is getting enough calories and nitrogen and is in an anabolic phase (21). ASPEN-FELANPE Clinical Guidelines recommend to provide protein and energy intake at a rate of 1.5–2.0 g/kg/d for adult ECF patients. Patients with EAF and high output ECF may require more protein (up to 2.5 g/kg/d) (23).

## Complications of TPN

However, the adverse effects of TPN including hyperglycemia, catheter-related bloodstream infection, central vein thrombosis, TPN-associated liver disease, and Refeeding Syndrome. TPN has negative effect on adaptive immunity, possibly due to reduced production of immunoglobulin A and gut-associated lymphoid tissue lymphocytes. However, TPN supplemented with glutamine improves innate immunity and can be used to resist bacterial mucosal invasion (24). Nowadays, TPN can be implemented much safer and it greater use is encouraged in those patients for whom the feasibility of providing EN is impossible (25, 26). PN should not be initiated for patients at low nutritional risk within the first 7 days, because of increased infection complications and mortality (27, 28).

## Refeeding Syndrome

Refeeding syndrome (RFS) is a serious complication, which is characterized by severe electrolyte and fluid shifts, vitamin deficiency and salt retention. RFS occurs in patients receiving nutritional support after severe malnutrition. When a patient who had been previously starved and malnourished is re-fed with a nutrient-dense diet, the absorbed plasma glucose and amino acids result in increased insulin secretion and decreased glucagon secretion. Insulin stimulates the absorption of potassium, magnesium and phosphate into cells. Subsequently, decreased serum phosphate, potassium and magnesium levels lead to the clinical features of RFS (29, 30).

When ECF occurs, many doctors used prolonged intravenous fluid repletion through the peripheral venous route, which cannot tolerate the hyperosmotic fluids of TPN. Therefore, many patients developed iatrogenic malnutrition due to insufficient calorie intake. When they were given regular nutrition support, many of them would present with RFS. Severe hypophosphatemia is a predominant feature of this syndrome. Supplementation with electrolytes (especially phosphates) and vitamins is the focus point of the treatment of ECF patients with RFS (31). Enteral refeeding syndrome, a subtype of RFS, occurs in ECF patients undergoing enteral feeding. Gut mucosal barrier dysfunction can develop in ECF patients due to lack of lumen nutrition. Once enteral feeding is carried out, intestinal motility and intraluminal nutrient load will increase. Bacteria or endotoxin will enter the bloodstream throughout discruped mucosal epithelium or loosened tight junctions. Continuous enteral feeding is the only solution for the problem (32).

Although RFS can cause severe complications, most physicians do not understand it well (33). Standardizing multidisciplinary nutrition care plans for patients with RFS can potentially reduce the incidence of complications (34). The 2016 ASPEN guidelines recommend that during the first week of ICU admission, the PN dose for severely malnourished patients should <20 kcal/kg day or 80% of estimated energy requirements (27). Only 1/3 to 1/2 of the caloric ration should be administrated on the first day. The concentration and dose should be gradually increase to the full amount of requirements. Vitamin C, electrolyte and trace elements such as zinc supplementation are also important for ECF treatment.

## PN Combined With EN

The benefits of enteral feeding in maintaining gastrointestinal mucosal health have been demonstrated. Compared with TPN, EN can reduce the risk of infection and related costs (35). Most centers use a combination of PN and EN (36, 37). Of a series of 1,168 patients at the Nanjing Fistula Treatment Center of Jingling Hospital, 75.9% received PN combined with EN, and 13.6% received PN only. The overall recovery rate reached 93% (37).

## EN and ECF

“If the gut works, use it or prepare to lose it (38).” In critically ill surgical patients, EN may have considerable advantages over PN. Compared with PN, EN is considered to improve intestinal barrier function, reduce the incidence of infection complications in critically ill patients, and maintain immune function (25, 39, 40). EN was identified as an independent factor associated with fistula closure (41). Therefore, more clinicians are attempting to perform EN in ECF patients after the initial phase of stabilization nowadays. Early enteral glutamine supplementation resulted in decreased intestinal permeability and gastrointestinal complications (42). However, glutamine supplementation in either PN or EN is a controversial topic. Martinez et al. (43) suggests that oral administration of arginine and glutamine supplementation 1 week prior to surgery may be beneficial for patients undergoing final surgery.

EN is believed to enhance the functional and structural integrity of the gastrointestinal tract. Further, EN not only prevents bacteria from adhering to intestinal epithelial cells, but also stimulates the secretion of immunoglobulin A and support the mass of gut-associated lymphoid tissue (GALT) (44, 45). However, EN is not usually recommended for high-output intestinal fistulas, especially when the output is more than 1.5 L/day. This is because EN may increase the amount of gastrointestinal secretion, which in turn increases the fistula output and thereby worsens malnutrition and delays fistula healing. If enteral feeding is insufficient to keep up with the high output or if nutritional requirements are not being met, then PN might be indicated. ASPEN-FELANPE Clinical Guidelines point out that EN may be feasible and tolerable in low output (<500 ml/d) ECF patients (suggesting no distal obstruction). However, those patients with high output (>500 ml/d) may require PN to meet fluid, electrolyte and nutritional requirements (28).

The mortality rate of high output EAFs is as high as 30%, while that of low output EAFs is 6% (46). EAF presenting in an open abdomen is now common and represents substantial challenges in nutritional support. Up to now, there are few studies on EN and EAF. Specifically, early EN should be attempted as the initial nutritional treatment for EAF patients, except for those patients aggravated by shock or severe intestinal obstruction after EN performed. Early EN could be successfully delivered for EAF patients after an open abdomen, with improved mortality risk (47). A retrospective study by Reinisch et al. (48) suggested that EN would not aggravate the wound status of OA/EAF patients. There was no significant increase in median fistula volume after EN initiation. Yin et al. (49) study demonstrated that EN could be safely implemented in patients in EAF patients without complicating the treatment of EAF. However, establishing an enteral feeding pathway in EAF patients may be difficult. Possible routes of EN include fistuloclysis, bypass of the fistula, and feeding jejunostomy.

Once the fistula anatomy is determined and EN is possible for the ECF patient, EN should be attempted immediately. Conditions for EN implementation includes intestinal continuity and sufficient length (usually more than 75 cm) of usable bower. Acute gastrointestinal injury (AGI) often occurs in patients with ECF. The Working Group on Abdominal Problems (WGAP) of the European Society of Intensive Care Medicine (ESICM) recommends starting a minimum EN (20 ml/h) within 24–48 h for AGI grade I patients. For patients with AGI grade II or III, it is recommended to initiate EN (20 ml/h) as well as other treatments based on the symptoms. For patients with AGI grade IV, EN is not recommended because those patients cannot tolerate EN (50, 51). According to the patient's ability to tolerate feeding, the maximum infusion rate can reach 120 ml/h per day. It is also important to avoid constipation and distal intestinal obstruction. Therefore, a balance must be achieved between slowing fistula output and avoiding constipation.

## Complications of EN

EN is generally safe and tolerable. Gastrointestinal complications, mechanical complications, infectious complications, and metabolic complication sometimes occur. Gastrointestinal complications such as diarrhea, nausea, and vomiting are frequent. During EN implement, infectious complications including pneumonia and bacterial contamination may happen. Furthermore, feeding tubes may be dislocate or cause perforation and gastrointestinal bleeding. RFS also occurs during enteral feeding but with lower incidence and severity compares to PN. However, careful nursing and monitoring can help reduce the incidence of complications (52).

## How to Establish EN Pathway?

Establishing EN pathway in ECF patients can be difficult and requires imagination and teamwork. Due to disturbed intestinal integrity of patients with ECF, it is impossible to use nasogastric tube as usual. Therefore, a variety of methods of EN have been developed (1). Nasointestinal tube can be used in upper fistula such as duodenal fistula and the tip of catheter can be placed below the fistula; (2). Intestinal fluid from the proximal fistula can be collected and then infused from the distal intestinal catheter in lower fistula (fistuloclysis) (53); (3). EN can still be carried out when the fistula is located very low, however drainage around the fistula should be well-established and short peptide be chosen for the formula of EN; (4). For multiple fistulas, the perfusion can be designed according to the site of the fistula. For example, intestinal fluid was collected from fistula 1, mixed nutrient solution was infused from fistula 2, then from fistula 3, fistula 4, which became relay perfusion (54). (5). ECF/EAF was occluded by special device and then EN was perfused (55). Those above should be conducted after some time when edema regression, because tissues are edematous and perforation could easily occur in acute phase.

## Fistuloclysis

**Figure 6:**
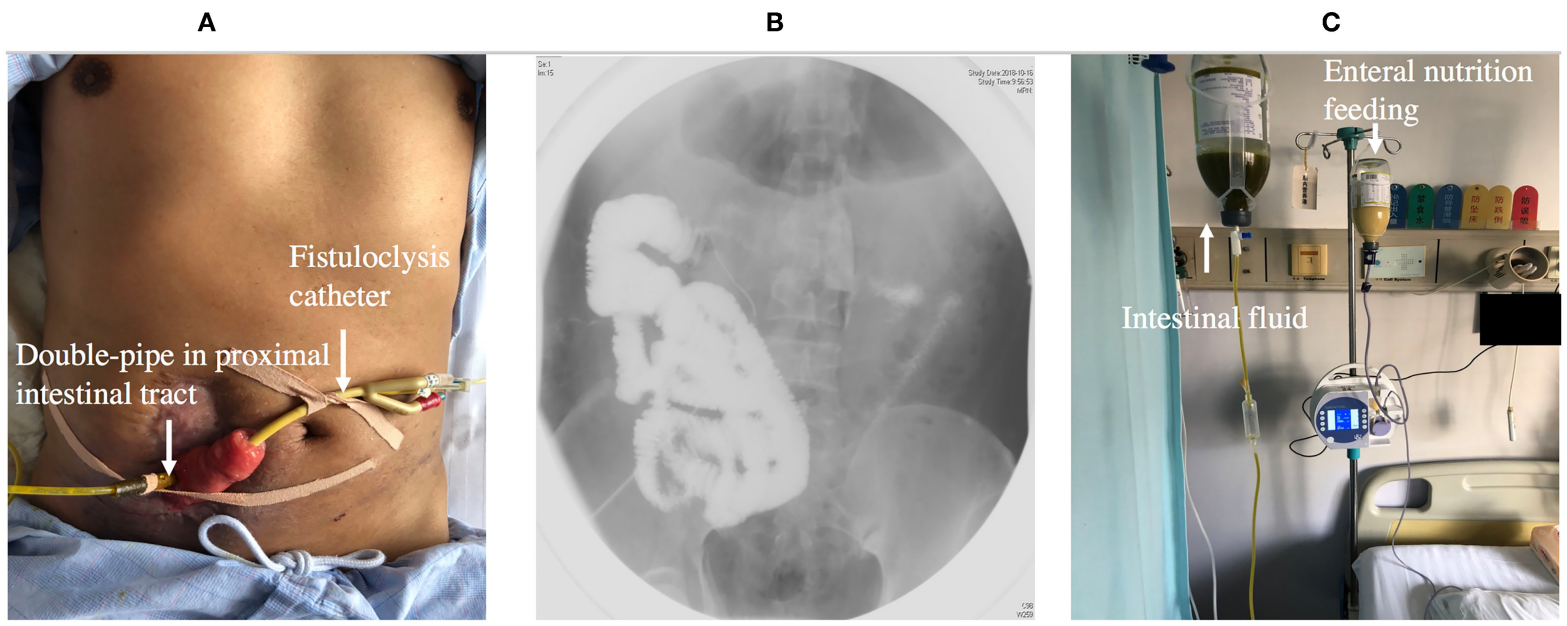
**(A)** Showing fistuloclysis: a double-pipe was intubated to collect intestinal fluid and a catheter was advanced to the lumen of the distal intestine for distal feeding. **(B)** Showing fistulogram before the start of fistuloclysis. **(C)** Showing that collected intestinal fluid was infused into distal intestinal lumen.

Fistuloclysis is an option that can provide nutritional support for those fistulas that cannot close spontaneously (56, 57). After completing the investigation of intestinal integrity and length of the small intestine beyond the distal fistula, a balloon Foley catheter was intubated into the fistula. Under radiological control, the catheter was pushed to a depth of 5 cm in the distal intestinal lumen and 5 ml of water was placed in the balloon of the catheter. The proximal intestine lumen was intubated with a double-pipe to collect intestinal fluid (Figure 6). Fistulolysis has proven to be a cost-effective alternative to TPN and can also stimulate the gut mucosa. The use of polymeric feed and elemental feed can provide the effective nutritional support for ECF patients (58).

## Fibrin Glue Sealing

**Figure 7:**
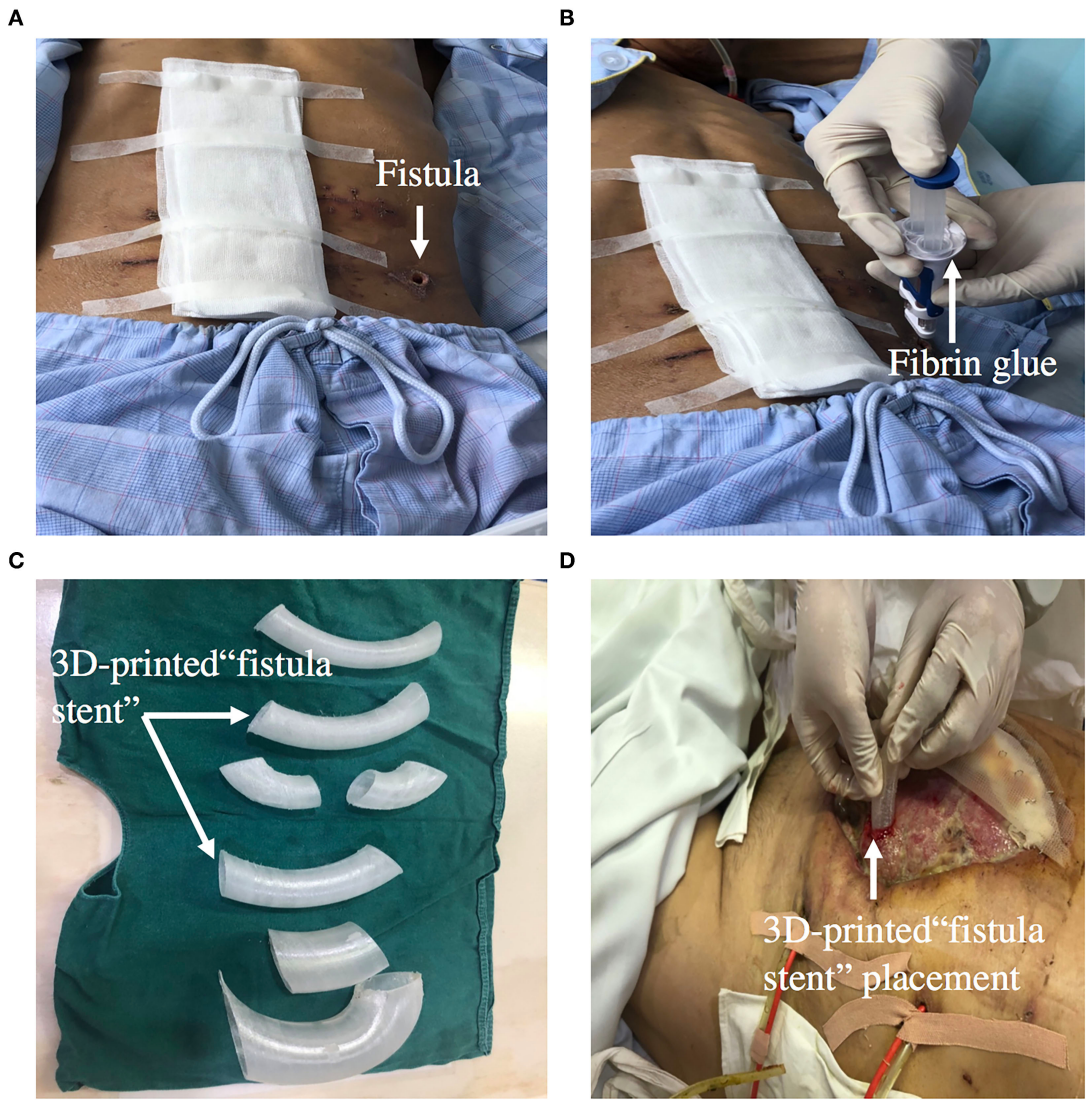
**(A,B)** Showing that ECF was occluded by fibrin glue. **(C,D)** Showing that 3D-printed “fistula stent” was implanted to reduce the volume of EAF effluent in the early stage of open abdomen.

Fibrin glue sealing has become an alternative option for occlusion of ECF because of it minimal invasiveness and simplicity. An observational cohort study reported that patients in the platelet-rich fibrin glue group had lesser median time of fistula closure than the control group (59). Furthermore, a multicenter, randomized, controlled clinical trial was designed to evaluate the glue application in the treatment of patients with low-output volume ECFs. The primary outcome of the trial is fistula closure time during the 14-day treatment period, which defined as the interval between the day of enrollment and day of fistula closure (60). Ren et al. reported that on the 9th day after fibrin glue application, enteral nutrition was successfully supplied and became the main nutrition therapy (61). Fibrin glue application should be used in patients with the following condition: the length of the sinus is longer than 2 cm, the diameter of the fistula is <1 cm, there is no pus in the sinus, and the fistula is a tubular fistula (Figures 7A,B).

## OTSC

Successful OTSC (Over-The-Scope Clip) placement in gastrointestinal fistula patients was reported by Hideki et al. (62). A total of 1,517 cases were described between 2010 and 2018 and the clinical success rate were calculated. The mean clinical success rate was 78% (*n* = 1517) and 52% of fistula (*n* = 388) (62). Law et al. (63) reported 47 patients underwent 60 operations using OTSC to close gastrointestinal fistulas. Initial technical success occurred in 41/47 (87%) cases. However, 19/41 (46%) patients experienced a recurrence of the fistula at a median of 39 days. The clinical success can be achieved only for relatively small fistulas <10 mm in size using a single OTSC (64). Roy et al. reported that 90% (9/10) of OTSC applications were technically successful and the overall success rate for ECF closure was 70% (65).

## 3D-Printed Personalized Fistula Stent

A 3D-printed personalized fistula stent for ECF treatment was reported by Jinling hospital. The stent was well-implanted and can effectively reduce the volume of ECF effluent (66). This 3D-printed fistula stent can be implanted in the early stage of open abdomen to close the EAF. It also reduces the fistula effluent, avoids the imbalance of water and electrolytes, and free from superficial and intraperitoneal infections. The patient's EN by nasal feeding was restored 4 days after implantation, which was started from 500 mL and increased to 1,500 mL during the following 3 d (67). Application of this novel “fistula stent” greatly accelerated rehabilitation processes (Figures 7C,D).

## Fistula Recurrence

Kluciński et al. (68) study showed that multiple fistulas, higher C-reactive protein level, and longer time interval from admission to definitive surgery were associated with an increased risk of severe complications or fistula recurrence. However, only multiple fistulas were an independent risk factor for severe complications or fistula recurrence in multivariate analysis. Those patients with high output, EAF, and/or history of open abdomen have the highest risk of recurrence after definitive surgery. Surgical interventions should be carried out in patients with optimal conditions without sepsis. The presence of sepsis is associated with higher mortality. Postponed surgery for ECF is associated with lower recurrence. Waiting a longer period can bring benefits such as improving fluids and electrolytes (69, 70). TPN should be used in those patients with high-output fistula to maintain nutritional status and reduce output. Pre-operative and post-operative use of TPN did not influence recurrence rate by univariate or multivariate analysis (71).

## Conclusions

Nutrition support plays a vital role in the management and successful closure of ECF. The principle of nutritional support for patients with ECF should be giving enteral nutrition priority, supplemented by parenteral nutrition. The basic principle of non-surgical treatment is to prevent further complications of ECF, such as sepsis, electrolyte imbalance, dehydration, and malnutrition. For high output ECFs, TPN is sometimes advocated to facilitate healing and reduce output. For low output ECFs, EN should be considered if the gut works. A variety of methods of enteral nutrition have been developed, such as fistuloclysis and relay perfusion. ECF could also be occluded by special device and then enteral nutrition was perfused, such as fibrin glue, OTSC, and 3D-printed patient-personalized fistula stent application. The following patients should be considered for definitive surgery: (1) eversion of the mucosa of the fistula; (2) the fistula has not closed spontaneous within 30 days; (3) those conditions exist such as distal obstruction, inflammatory bowel disease, neoplasm, radiation enteritis.

With the progress of anti-infection, nutritional support and definitive treatment of ECF, the prognosis of ECF has been significantly improved. However, further prevention of its occurrence, shortening the treatment course and reduction of complications are still the directions of future research.

This is a narrative review on ECFs and intestinal failure and the best way to approach this clinical condition form a clinical nutrition aspect. This review also includes some new interesting information including the information about the stent and the other technique for closing fistulas. The limitation of this review is that its focus is too narrow to be a comprehensive review.

## Ethics Statement

Written informed consent was obtained from the individuals for the publication of any potentially identifiable images or data included in this article.

## Author Contributions

J-aR and J-sL equally contributed to the conception and design of the research. Z-wH, H-jR, and LW contributed to the design of the research. G-fW, G-sG, and JC contributed to the acquisition and analysis of the data. TZ contributed to the interpretation of the data. X-wW and Q-qT drafted the manuscript. All authors critically revised the manuscript, agree to be fully accountable for ensuring the integrity and accuracy of the work, and read and approved the final manuscript.

## Conflict of Interest

The authors declare that the research was conducted in the absence of any commercial or financial relationships that could be construed as a potential conflict of interest.
